# Phenomenology and Digital Knowledge: Introduction to the Special Issue

**DOI:** 10.1007/s10699-023-09905-0

**Published:** 2023-02-23

**Authors:** Floriana Ferro, Luca Taddio

**Affiliations:** University of Udine, Udine, Italy

This Special Issue takes inspiration from the debate occurred during the international conference “Phenomenology and Digital Knowledge—Fenomenologia e saperi digitali”, which took place in Udine from September 1 to 4, 2021. Some of the participants in the conference have developed their reflections in writing, thus realizing their contribution for this issue. The debate is further enriched by other authors of international relevance, who have decided to participate in the realization of this volume. The main question directed to the authors of the issue was the following: what is the relevance of phenomenology and its theoretical value today?

The aim of this volume is to consider the multiple intersections between phenomenology and scientific investigation, placed between neurosciences and complexity theory. The field of investigation is particularly defined by the phenomenology of perception in relation to the ongoing processes of transformation involving the digital, artificial intelligence, and hybridization. The issue hosts a discussion and a deepening of the general theories of perception, especially visual one, the experimental aspects of virtual objects and digital environments, the common architectural space between human and non-human, as well as the theoretical implications on aesthetics and ontology. These relations and the multiplicity of digital dimensions open to the possibility of rethinking and modifying the human. The aim is to understand deeply the relevance of phenomenology and its ability to interpret changes in the contemporary world. However, other contributions coming from different perspectives, which are critical towards phenomenology, have enriched the philosophical debate in this volume.

Five contributions of the issue are focused on digital objects, relations and bodies, and on how they can be phenomenologically conceived. In Taddio’s paper, entitled “From Perception to the Digital World: Phenomenological Observations,” the concept of illusion in digital environments and immersive virtual realities is developed. The author argues, through his background in experimental phenomenology and Gibson’s ecological perspective, how Merleau-Ponty’s concept of “incarnate” is related to the more general concept of “illusion.” In order to understand the importance of the experiential process and its irreducibility to the scientific model of reality, Taddio focuses on specific cases of optical-geometric illusions (i.e., Müller-Lyer, stroboscopic movement, Gelb effect, etc.) and their ontological and epistemological implications. These implications are then applied to the digital world and to the possibilities of hybridization between our body and the digital dimension.

Ferro’s article, “Perceptual Relations in Digital Environments,” discusses the issue of perception of objects in both analog and digital dimensions from a phenomenological perspective. The author particularly analyzes the concept of “perceptual relation,” conceived by the founders of Gestalt psychology as the interaction between the whole and its parts. Ferro reconfigures this idea by challenging the Gestaltist idea of intrinsic relation and arguing that it is extended to the surrounding *Umwelt*. For this purpose, she analyzes our perception of the relation between the whole and its parts in a specific object (a ball), located in four kinds of digital environments (on-screen, virtual, augmented, and hybrid). When the environment changes, some modes of appearance of the object (multisensoriality, figure-ground interaction, affordances, and persistence) are modified, but the general whole-part relation remains by virtue of a transdimensional analogy.

Harman’s “Merely Intentional Objects: A Defense” does not embrace a phenomenological perspective, but offers an alternative view, which is based on Object-Oriented Ontology (OOO). The author starts from Husserl’s early paper “Intentional Objects” and confutes his idea that there are not merely intentional objects, because thinking to a non-existent object means thinking only to qualities without an object. Harman argues that Husserlian phenomenology implies that an object-pole is always there, even in case of imagination or hallucination. Harman further develops his argument, pointing out that Husserl already shows a theory of the quadruple object: this makes him an illustrious predecessor of OOO and opens the way to the theorization of digital objects, whose mode of existence resembles the merely intentional objects.

Osler and Zahavi, in their paper entitled “Sociality and embodiment: online communication during and after Covid-19,” particularly focus on human relations and how they differ when they take place in online platforms. The authors take inspiration from the phenomenology of sociality, considering the impact of digitally-enabled forms of communication and sociality on our experience of the other. Osler and Zahavi argue, through the analysis of perceptual access, intercorporeality, shared space, transitional spaces, and self-presentation, that our embodied experience of the other is definitely altered and presents clear differences with face-to-face encounters. This alteration points out how digital mediation affects our communication and why it cannot be thought of in terms of replacement.

Cantone, in his “The simulated body. A preliminary investigation into the relationship between Neuroscientific studies, Phenomenology and Virtual Reality,” compares immersive VR technology, phenomenology and Neural Darwinism in order to discuss the issue of the simulated body, which is not one’s own body or exists in a different perceptual configuration. Cantone argues that Virtual Reality seems to partially replicate the simulation mechanisms of our mind, thus allowing the simulated body to get closer to our “fleshy” body in analog reality.

Three papers of the issue point out foundational problems about phenomenology and digital knowledge. Tagliagambe’s *Phenomenology and the Digital World: Problems and Perspectives* analyzes Husserlian division between passive synthesis, regarding the issue of genesis, and active and vigilant processes. The problem of consciousness as anoetic, noetic, and autonoetic is considered, shedding new light on the mind-body problem and the most recent achievements in neuroscience, especially in relation to the digital evolution.

Lanfredini, in “Digital and analogue Phenomenology,” draws a distinction between two ways of conceiving phenomenology, arguing for a digital reading of it which does not contrast its fundamental way of thinking. In order to reach this aim, Lanfredini shows that static phenomenology may be also considered as digital, since is it based on the concepts of determination or datum and of extensive quality. On the other hand, analogue phenomenology is compared to Husserl’s genetic approach and Merleau-Ponty’s phenomenology of perception, which is based on the idea of tension or force and, consequently, on intensive or forceful quality. These two ways of performing phenomenological description are both fundamental, so that the digital approach is legitimate and allows to investigate properly the products of the digital revolution.

Zhok, in his “Phenomenology and complexity,” reads our reality through the lenses of the theory of complexity, performing a phenomenological analysis on it. He shows that efficient causality is not sufficient to explain reality, may it be digital or analog, and argues for a different ontology, according to which there are qualities irreducible to deduction and emerging from existing qualities. Whereas reductionist models turn out to be insufficient, phenomenology and complexity can account for this kind of qualities and related phenomena.

Four papers of this issue are particularly focused on Artificial Intelligence and algorithmic thinking. Buongiorno, in her “Can algorithms be embodied? A phenomenological perspective on the relationship between algorithmic thinking and the life-world,” discusses the difference between artificial and human intelligence from a phenomenological perspective. She argues that this difference has to be reconsidered, since digital processes do not imply either a detachment from the body or dematerialization. In order to reach this aim, Buongiorno refers to cyberpunk narratives and develops a phenomenological analysis which relies on Hayles’ distinction between incorporating and inscribing practices, to Maturana and Varela’s idea of structural coupling, and to the temporal structure of algorithmic thinking.

Possati’s paper, “From Turing to Peirce. A semiotic interpretation of computation,” argues that artificial intelligence based on computational systems includes semiotic processes and is thus similar to human intelligence. The author shows that semiotic relations are essential to computation and, for this purpose, he re-interprets the Turing machine through the recourse to Peirce’s semiotics; moreover, he confutes the reductionist and Piccinini’s mechanicist model of physical computation, thus showing that semiotic processes are implied in computational systems.

In “Philosophical, Experimental and Synthetic Phenomenology. The Study of Perception for Biological, Artificial Agents and Environments,” Calì discusses how the model proposed by synthetic phenomenology, which was mainly developed in the first decade of the 20th century, addresses the question of artificial agents and of their phenomenal access to the environment. He shows some examples of architectures and models for artificial agents with phenomenal states offered by synthetic phenomenology and argues that the phenomenology of perception, in both its Husserlian and Gestaltist versions, makes a relevant contribution to synthetic phenomenology.

Cusano’s “Cobot and Sobot: For a new ontology of collaborative and social robot” offers an Aristotelian interpretation of the development of robots which openly interact with humans. The latter can be generally defined as “cosbots”, since they are either cobots (collaborative robots) or sobots (social robots). The author argues that the cobot is social only in potency, whereas the sobot is social in act. This Aristotelian distinction classifies robots according to the degree of development of artificial intelligence, and opens to the possibility of encouraging machine self-learning, since autonomy is considered as the condition of sociality.

